# Applying Clinical Decision Support Design Best Practices With the Practical Robust Implementation and Sustainability Model Versus Reliance on Commercially Available Clinical Decision Support Tools: Randomized Controlled Trial

**DOI:** 10.2196/24359

**Published:** 2021-03-22

**Authors:** Katy E Trinkley, Miranda E Kroehl, Michael G Kahn, Larry A Allen, Tellen D Bennett, Gary Hale, Heather Haugen, Simeon Heckman, David P Kao, Janet Kim, Daniel M Matlock, Daniel C Malone, Robert L Page 2nd, Jessica Stine, Krithika Suresh, Lauren Wells, Chen-Tan Lin

**Affiliations:** 1 Department of Clinical Pharmacy Skaggs School of Pharmacy and Pharmaceutical Sciences University of Colorado Anschutz Medical Campus Aurora, CO United States; 2 Adult and Child Consortium for Outcomes Research and Delivery Science University of Colorado Aurora, CO United States; 3 Department of Clinical Informatics University of Colorado Health Aurora, CO United States; 4 Department of Medicine School of Medicine University of Colorado Anschutz Medical Campus Aurora, CO United States; 5 Charter Communications Corporation Greenwood Village, CO United States; 6 Section of Informatics and Data Science, Department of Pediatrics School of Medicine University of Colorado Anschutz Medical Campus Aurora, CO United States; 7 University of Colorado Clinical and Translational Sciences Institute Aurora, CO United States; 8 VA Eastern Colorado Geriastric Research Education and Clinical Center Aurora, CO United States; 9 Department of Pharmacotherapy Skaggs College of Pharmacy University of Utah Salt Lake City, UT United States

**Keywords:** PRISM, implementation science, clinical decision support systems, RE-AIM, congestive heart failure

## Abstract

**Background:**

Limited consideration of clinical decision support (CDS) design best practices, such as a user-centered design, is often cited as a key barrier to CDS adoption and effectiveness. The application of CDS best practices is resource intensive; thus, institutions often rely on commercially available CDS tools that are created to meet the generalized needs of many institutions and are not user centered. Beyond resource availability, insufficient guidance on how to address key aspects of implementation, such as contextual factors, may also limit the application of CDS best practices. An implementation science (IS) framework could provide needed guidance and increase the reproducibility of CDS implementations.

**Objective:**

This study aims to compare the effectiveness of an enhanced CDS tool informed by CDS best practices and an IS framework with a generic, commercially available CDS tool.

**Methods:**

We conducted an explanatory sequential mixed methods study. An IS-enhanced and commercial CDS alert were compared in a cluster randomized trial across 28 primary care clinics. Both alerts aimed to improve beta-blocker prescribing for heart failure. The enhanced alert was informed by CDS best practices and the Practical, Robust, Implementation, and Sustainability Model (PRISM) IS framework, whereas the commercial alert followed vendor-supplied specifications. Following PRISM, the enhanced alert was informed by iterative, multilevel stakeholder input and the dynamic interactions of the internal and external environment. Outcomes aligned with PRISM’s evaluation measures, including patient reach, clinician adoption, and changes in prescribing behavior. Clinicians exposed to each alert were interviewed to identify design features that might influence adoption. The interviews were analyzed using a thematic approach.

**Results:**

Between March 15 and August 23, 2019, the enhanced alert fired for 61 patients (106 alerts, 87 clinicians) and the commercial alert fired for 26 patients (59 alerts, 31 clinicians). The adoption and effectiveness of the enhanced alert were significantly higher than those of the commercial alert (62% vs 29% alerts adopted, *P*<.001; 14% vs 0% changed prescribing, *P*=.006). Of the 21 clinicians interviewed, most stated that they preferred the enhanced alert.

**Conclusions:**

The results of this study suggest that applying CDS best practices with an IS framework to create CDS tools improves implementation success compared with a commercially available tool.

**Trial Registration:**

ClinicalTrials.gov NCT04028557; http://clinicaltrials.gov/ct2/show/NCT04028557

## Introduction

### Background and Significance

Clinical decision support (CDS) tools within electronic health records (EHRs) hold the promise of improved patient care, but they are not always effective. To optimize effectiveness, developers are encouraged to apply CDS design best practices (eg, user-centered design) [1-4]. However, the comprehensive application of CDS best practices is resource intensive, and health care institutions are faced with an ever-growing list of CDS development projects. With limited resources, institutions often rely on commercially available CDS tools, which generally require fewer resources for deployment. Commercial CDS tools are created to meet the generalized needs of many institutions and thus may not integrate well into institution-specific workflows. Designing for the generalized needs of many institutions is not user centered. Thus, it violates a key CDS design best practice principle. Some have also asserted that commercial CDS tools may be based on content knowledge systems that are uninformative and not clinically relevant [1,5]; thus, they are less likely to be adopted [5,6]. However, these assertions have not been tested.

Although retrospective studies suggest that CDS best practices may improve CDS effectiveness [2-4,7,8], they are often minimally applied. Beyond resource availability, reasons for their minimal application may include skepticism about the evidence and insufficient guidance on how to apply them. Although CDS best practices acknowledge the importance of thoughtful implementation, they do not provide clear guidance regarding implementation considerations. Therefore, integration with evidence-based implementation science (IS) frameworks such as the Practical, Robust, Implementation, and Sustainability Model (PRISM) [9] can provide the direction needed to comprehensively apply CDS design best practices [10]. Such an integrated approach accounts for the many contextual factors that influence implementation success and makes CDS implementation more replicable. To maximize the quality of patient care, institutions need to understand the return on investment from allocating resources to apply CDS design best practices compared with relying on commercially available CDS tools.

### Objective

The objective of this study is to compare the effectiveness of an enhanced CDS tool informed by CDS design best practices and the PRISM IS framework with a prepackaged, commercially available CDS tool. The use case for this evaluation was an evidence-based beta-blocker (BB; bisoprolol, carvedilol, and metoprolol succinate) prescribed for patients with heart failure with reduced ejection fraction (HFrEF) in primary care. This use case was selected because it represents a national guideline recommendation with suboptimal adherence and both clear and compelling patient care implications [11-15]. Our hypothesis was that the enhanced CDS tool would result in greater clinician adoption and be more effective in changing prescribing than the commercial CDS tool.

## Methods

### Study Design

**Figure 1:**
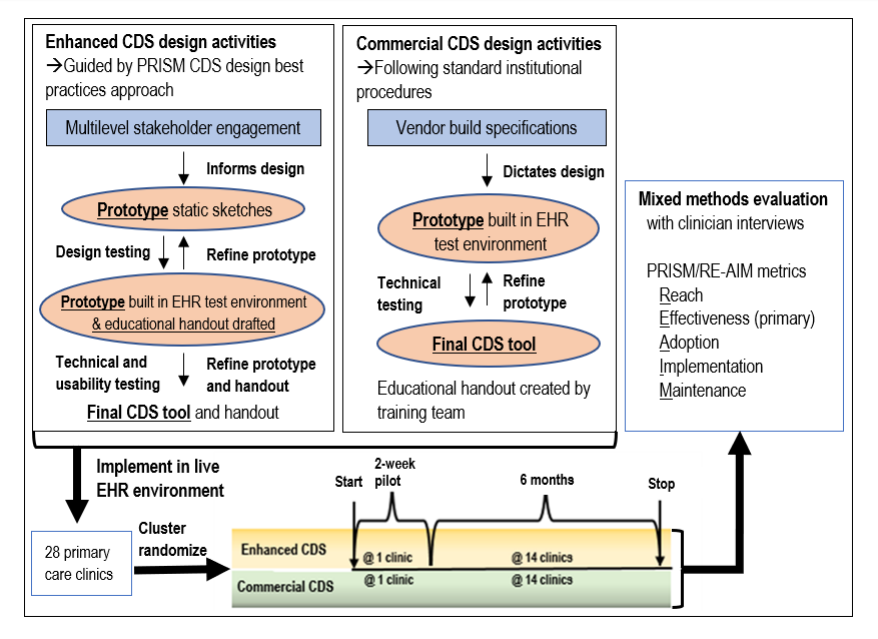
Study design overview.

We conducted an explanatory sequential mixed methods study [16] at UCHealth, a large regional health system representing more than 5 million unique patients across diverse clinical settings. Since 2011, UCHealth has used the Epic EHR software program (Epic Systems). The first study phase was a cluster randomized controlled trial (RCT; NCT04028557), and the second phase consisted of a series of qualitative interviews with clinicians. Both phases were guided by the PRISM framework. Figure 1 provides an overview of the study design. The study design and reporting were guided by the CONSORT (Consolidated Standards of Reporting Trials) and best practices in complex trial interventions [17-19]. The study was approved by the Colorado Multiple Institutional Review Board.

### Description of the CDS Interventions

**Figure 2:**
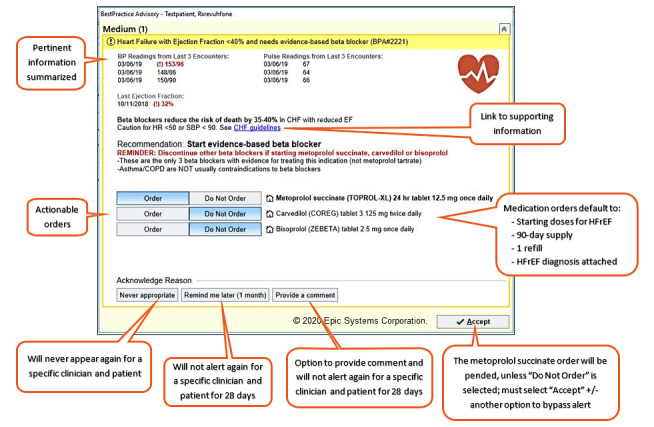
Representative user interfaces of the enhanced clinical decision support alerts.

**Figure 3:**
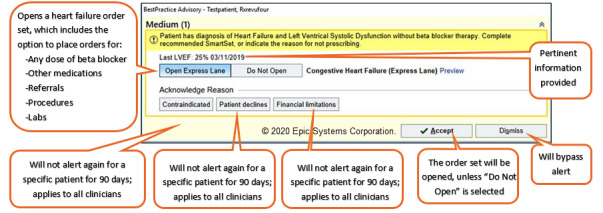
Representative user interfaces of the commercial clinical decision support alerts.

We evaluated 2 CDS tools within the EHR: a commercial alert and an enhanced alert. The automated alerts interrupted primary care providers (PCPs) when they opened a patient’s chart during an office visit if the patient had a diagnosis of HFrEF and had not been prescribed evidence-based BB therapy. The CDS referred to the most recent ejection fraction (EF) value from an echocardiogram and/or a diagnosis of interest. Table 1 describes the build specifications and compares the way in which each CDS tool identifies an HFrEF diagnosis. Figures 2 and 3 depict the user interface for the enhanced and commercial CDS tools, respectively. Both alerts used the EHR-native CDS software *BestPractice Advisory* and underwent technical testing in EHR test environments. A 1-page educational handout on each alert was shared with clinician end users at the discretion of their respective clinic leaders or managers. There were some distinct differences in the design and implementation activities of each alert.

**Table 1:** Summary of build specifications for the enhanced and commercial alerts^a^.

Enhanced CDS^b^		Commercial CDS
**Inclusion criteria**		
	≥18 years old A diagnosis that explicitly states an EF^c^ ≤40% or an echocardiogram result indicating EF≤40%	≥18 years old Any HF^d^ diagnosis and an echocardiogram result indicating EF≤40%
**Exclusion criteria**		
	Prescribed or pended order for metoprolol succinate, carvedilol, or bisoprolol. Relied on knowledge management customized to the institution BB^e^ allergy using knowledge management customized to the institution	Prescribed some versions of metoprolol tartrate, metoprolol succinate, carvedilol, or bisoprolol. Relied on vendor-supplied knowledge management, which did not comprehensively represent these BBs BB or beta-agonist allergy using vendor-supplied knowledge management
**Recommended action**		
	Can pend evidence-based medication orders at starting doses without leaving the UI^f^	Can open order set from UI, which opens a new screen and provides option to order any dose of BB, other drugs, labs, echo, and schedule follow-up visits
**Response options (acknowledge reasons)**		
	Options: Never appropriate, remind me later (1 month), provide comment When a user selects a response option other than “never appropriate,” it will not alert again for that user and patient for 28 days. If a user selects “never appropriate,” it will not alert for that user and patient for >20 years No dismiss button	Options: Contraindicated, cost concern, patient declines When a user selects a response option, it will not alert again for any user for that patient visit for 90 days Dismiss button option
**How to close**		
	Easiest way to dismiss is to hit accept, which pends order for metoprolol succinate Must select 1 of 3 acknowledge reasons or pend order for 1 of the BB options in the UI	Must select “dismiss,” open order set, or select 1 of 3 acknowledge reasons in the UI
**Pertinent information displayed**		
	Patient has HF and reduced EF BB indicated Values: most recent EF, last 3 BP^g^ and HR^h^ measurements Benefit of starting BB—longevity Parameters for caution: HR<50 and BP<90/60 Asthma and chronic obstructive pulmonary disease are not contraindicated Metoprolol tartrate is not evidence-based Reminder to discontinue other BBs Link to supporting reference	Patient has HF and reduced EF BB indicated Values: most recent EF
**Trigger**		
	Open patient visit or encounter	Open patient visit or encounter
**Other features**		
	Abnormal values of BP, HR, and EF are emphasized in red font	None to note

^a^Key differences are italicized.

^b^CDS: clinical decision support.

^c^EF: ejection fraction.

^d^HF: heart failure.

^e^BB: beta-blocker.

^f^UI: user interface.

^g^BP: blood pressure.

^h^HR: heart rate.

### Enhanced CDS Alert

We designed and implemented the enhanced alert by applying the PRISM/CDS best practices approach, as previously described [10]. Briefly, PRISM is an expanded version of the widely used Reach, Effectiveness, Adoption, Implementation, and Maintenance (RE-AIM) measures [20,21] that includes additional contextual factors that influence implementation success [9]. The integrated PRISM/CDS best practices approach incorporates an iterative, user-centered design process with 5 phases: (1) multilevel stakeholder engagement, (2) designing the CDS tool, (3) design and usability testing, (4) thoughtful deployment, and (5) performance evaluation and maintenance [10]. Following PRISM, this approach considers the dynamic interactions between the internal and external environments [9]. We solicited extensive stakeholder input from clinicians [22] and patients to understand their needs, preferences, and values for the CDS design and treatment recommendations. Stakeholder input informed the enhanced alert design, which then underwent design and usability testing with clinicians. During usability testing, clinician end users refined the educational handout for the enhanced CDS alert.

### Commercial CDS Alert

The commercial alert served as the active control. The EHR vendor provided the build specifications that are available for use by all vendors’ institutions. To be consistent with the way in which commercial alerts are commonly used, the build specifications were not informed by the PRISM/CDS best practices approach and did not change based on stakeholder input. However, we modified the build specifications to align with evidence-based and institution-specific practices. No modifications were made that would bias the results against the commercial alert. Multimedia Appendix 1 outlines the changes made to the commercial build specifications and the rationale for the changes. An educational handout on the commercial CDS was created following standard institutional procedures, including a review by the institution’s dedicated training team.

### Phase 1: Cluster RCT

#### Study Design and Randomization

Both alerts were deployed across 28 UCHealth primary care clinics (2 geriatrics, 17 family medicine, and 9 internal medicine) using a modified randomized parallel group design. Each alert was piloted in 1 clinic for 2 weeks before widespread implementation to facilitate clinic buy-in (Figure 1). Our initial plan was to implement the alerts in parallel at mutually exclusive clinics for 6 months. However, an a priori planned interim analysis revealed no instances of the commercial alert changing prescribing. Therefore, we determined that there was no added benefit of the commercial alert and stopped the trial early.

We performed cluster randomization, in which the cluster was defined as the clinic. Block randomization was used to allocate 1 of the 2 alerts (commercial or enhanced) to each clinic, with 6 blocks or strata, defined by geographical location (ie, North, South, or Metro) and volume of HFrEF patients (ie, small or large) [23-25]. Small-volume clinics had fewer than 25 patients with HFrEF. We used a random sampling scheme (function *sample_n* in R statistical software) to randomly assign half of the clinics in each block to the commercial alert. Only the study investigators knew to which group each clinic had been assigned.

#### Study Population

All 28 clinics that agreed to participate were included in the study. Clinicians in the participating clinics were evaluated if they were ever exposed to 1 of the alerts. A patient’s EHR record was evaluated if 1 of the alerts was triggered.

#### Data Collection

Data were collected from the EHR via a CDS reporting analytic utility, chart review, and a secondary EHR virtual data warehouse. The EHR analytic utility allows postimplementation surveillance of CDS activity that includes when an alert is triggered, the identity of the patient and clinician, and the clinician’s response to the alert (buttons clicked). Chart reviews were used to verify clinician-stated responses to the alert. Data collected from the data warehouse included patient and clinician characteristics. Comorbidities of interest were collected starting on the first day the alerts were deployed and are described in Multimedia Appendix 2. Concurrent medications included those prescribed within 12 months before the first day of deployment.

#### Primary RE-AIM Outcome: Effectiveness

Effectiveness was measured as the proportion of alerts that resulted in an evidence-based BB prescription when indicated.

#### Secondary RE-AIM Outcomes: Safety, Reach, Adoption, Implementation, and Maintenance

Effectiveness was also balanced by a safety evaluation that identified instances of bradycardia (heart rate<50 bpm), hypotension (blood pressure [BP]<90/60 mmHg), acute heart failure exacerbation requiring hospitalization or an emergency department visit, and unintended consequences, such as duplicate therapy. We evaluated the safety outcomes during the 1-month period after each alert was triggered. Reach was measured as the number of alerts for unique patient visits, unique patients, and unique clinicians. We also measured reach as the proportion of unique alerts relative to the number of patients with HFrEF. We selected this denominator based on data availability to allow for comparisons of representativeness between the 2 alerts. Adoption was measured as the proportion of times a clinician responded to an alert and was stratified by unique patients and clinicians. Overall, an alert was classified as *adopted* when the clinician paid attention to the information presented and did something other than dismiss it outright. Multimedia Appendix 3 provides details of how we defined adoption. Implementation was assessed by documenting the types and number of changes to the alert design or workflow integration [21]. Maintenance was assessed based on whether the intervention continued after the trial ended.

#### Data Analysis

Differences in overall effectiveness and adoption rates based on the number of alerts were tested using the chi-square test of independence. We compared the baseline characteristics of patients using a 2-sample *t* test for continuous variables and a chi-square test for independence for categorical characteristics. For chi-square tests in which any cell count was less than 5, a simulated *P* value with 2000 simulation replicates was calculated. For all analyses, R statistical software was used, and *P*<.05 was considered statistically significant.

### Phase 2: Qualitative Interviews

#### Measures and Procedures

We invited clinicians exposed to 1 of the 2 alerts to participate in brief semistructured interviews. We used purposeful sampling to maximize the representativeness of exposure, practice setting, and clinician type. To capture the breadth of end user perspectives, interviews were conducted until saturation of ideas was reached. The participants provided informed consent. An investigator (KT) with domain expertise in primary care and CDS led each 30-min interview. The interviews followed a semistructured moderator guide, which was adapted as important concepts arose. Interviews were conducted in person or via a video conference. Audio recordings were transcribed and validated by independent investigators (JS and JK). Multiple strategies have been employed to maximize the rigor and quality of the methods and analysis [26], including triangulation, audit trails, and bracketing.

The interviews consisted of 3 consecutive components: (1) recall of the alert to which participants were exposed, (2) completion of the modified System Usability Scale (SUS) for that alert, and (3) introduction to the alert they had not been exposed, followed by a discussion comparing positive and negative features that influence the adoption of each alert. The validated SUS [27] terminology was modified to fit the HFrEF alert situation, and 2 questions were added to address ease of workflow integration and perceived impact on patient care.

#### Data Analysis

We used a thematic approach [28] and ATLAS.ti software (version 7, Scientific Software Development GmbH) to analyze the transcripts. A codebook was created a priori, which involved discussion among the 3 investigators (KT, JS, and DM). The changes to the codebook were documented. One investigator (JS) iteratively categorized the transcriptions into major themes that differentiated the 2 alerts using topic coding and analytical coding [29]. A second independent investigator (KT) reviewed the coding for the validation. Implementation measures of usability from the modified SUS survey were summarized according to the validated weighting system [27,30], and responses to the 2 additional questions were summarized descriptively.

## Results

### Patient Reach, Clinician Adoption, and Prescribing Effectiveness

The 2 alerts were deployed across 28 primary care clinics between March 15 and August 23, 2019. The mean age of patients exposed to an alert was 75.3 (SD 13.2) years, 70% (58/83) were male, and most were non-Hispanic and had Medicare insurance. Table 2 summarizes the characteristics of the patients who triggered the alerts.

The enhanced alert was triggered 106 times for 61 unique patients and 87 unique clinicians. The commercial alert was triggered 59 times for 26 unique patients and 31 unique clinicians. Patient visits were not always performed by the same clinician. Clinics allocated to the enhanced alert had 397 patients with HFrEF, compared with 307 patients in clinics allocated to the commercial alert; thus, reach was 26.7% (106/397) and 19.2% (59/307) for the 2 groups, respectively.

The overall adoption rate was significantly higher with the enhanced alert than with the commercial alert (62.3% vs 28.8%; *P*<.001). A total of 4 patients and 1 clinician were exposed to both commercial and enhanced alerts. None of these alerts led to a BB prescription, but adoption was higher with the enhanced alert. The 4 patients had a total of 7 visits with the enhanced alert, and 86% (6/7) resulted in adoption. The same 4 patients had a total of 12 visits with a commercial alert, and 2% (5/12) resulted in adoption. The single clinician who was exposed to both alerts adopted (did not outright dismiss) the enhanced alert each of the 2 times it was triggered (100%) and adopted the commercial alert 3 of the 6 times it was triggered (50%).

The enhanced alert was effective in changing prescribing for 15 of 61 unique patients (25%), whereas the commercial alert did not change prescribing at all. The overall rate of BB prescription was significantly higher when clinicians received the enhanced alert compared with the commercial alert (14.2% vs 0%; *P*=.006). Table 3 summarizes the results of the number of alerts, adoption, and effectiveness.

No adverse drug events were observed among patients who were prescribed a BB. When considering possible unintended consequences, a chart review revealed that 2 clinicians unintentionally ordered a BB from the customized alert, and the pharmacy processed the prescriptions, but neither patient picked up their prescription. These unintentional prescriptions were excluded from the effectiveness outcome. The enhanced alert was also triggered for 2 patients with a documented allergy to *beta-adrenergic blocking agts,* and neither led to a BB prescription. On the basis of clinician feedback, we identified and corrected an error in the build specification for the commercial alert during the first month of deployment; however, this error did not impact measures of reach, adoption, or effectiveness. Furthermore, upon completion of the study, the health system decided to continue the enhanced alert across all 28 clinics for operational and nonstudy purposes.

**Table 2:** Baseline characteristics of patients exposed to the alerts (N=83).

Characteristic	Enhanced alert (n=61)	Commercial alert (n=26)	Total^a^ (N=83)	*P* value
Age (years), mean (SD)	74.8 (12.8)	76.6 (15)	75.3 (13.2)	.16
Male, n (%)	40 (66)	19 (73)	58 (70)	.66
White, n (%)	57 (93)	22 (85)	75 (90)	.23
Hispanic, n (%)	5 (8)	2 (7)	7 (8)	.99
Medicare, n (%)	50 (82)	22 (85)	69 (83)	.99
Primary care provider type: attending physician, n (%)	39 (64)	25 (96)	63 (76)	.01
Left ventricular ejection fraction, mean (SD)	31.7 (11)	34.7 (6)	32.7 (9)	.11
Heart rate, mean (SD)	78.7 (17)	73.4 (15)	76.9 (17)	.16
Heart rate<50, n (%)	1 (2)	1 (4)	2 (2)	.99
Systolic blood pressure, mean (SD)	123.7 (18)	121.0 (18)	123.3 (18)	.51
Diastolic blood pressure, mean (SD)	70.0 (12)	71.3 (9)	70.5 (12)	.59
Blood pressure <90/60, n (%)	1 (2)	1 (4)	2 (2)	.99
≥1 visit with cards^b^ in past 1 year, n (%)	32 (53)	18 (69)	47 (57)	.23
≥1 visit with cards in past 2 years, n (%)	39 (64)	20 (77)	56 (68)	.35
Past BB^c^, ever, n (%)	49 (80)	18 (69)	64 (77)	.40
BB allergy per chart review^d^, n (%)	2 (3)	0 (0)	2 (2)	.59
BB intolerance or contraindication per chart review, n (%)	10 (16)	4 (15)	14 (17)	.99
Prescribed nonevidence-based BB^e^, n (%)	29 (48)	12 (46)	38 (46)	.99
Prescribed metoprolol tartrate, n (%)	22 (36)	8 (31)	28 (34)	.82
Prescribed angiotensin converting enzyme inhibitor or angiotensin receptor blocker or ARNI^f^, n (%)	37 (61)	18 (69)	55 (67)	.61
Prescribed ARNI, n (%)	1 (2)	0 (0)	1 (1)	.99
Prescribed mineralocorticoid receptor antagonist, n (%)	11 (18)	6 (23)	17 (20)	.80
Prescribed nondihydropyridine calcium channel blocker, n (%)	1 (2)	1 (4)	1 (1)	.99
Chronic obstructive pulmonary disease, n (%)	9 (15)	6 (23)	15 (18)	.53
Asthma, n (%)	7 (12)	3 (12)	10 (12)	.99
CAD^g^ (myocardial infarction, percutaneous coronary intervention, bypass, CAD, angioplasty), n (%)	34 (56)	14 (54)	48 (58)	.99
Nonischemic cardiomyopathy, n (%)	19 (31.1)	10 (38.5)	29 (34.9)	.68
Atrial fibrillation, n (%)	25 (41.0)	15 (57.7)	40 (48.2)	.23

^a^Four patients were exposed to both the enhanced and commercial CDS.

^b^cards: outpatient cardiology provider.

^c^BB: beta-blocker.

^d^These patients were inadvertently not excluded from the alert.

^e^Other nonevidence-based beta blockers included atenolol, nebivolol, and sotalol.

^f^ARNI: angiotensin receptor-neprilysin inhibitor.

^g^CAD: coronary artery disease.

**Table 3:** Description of clinical decision support alerts, adoption, and effectiveness.

Characteristics		Enhanced	Commercial
**Alerts for patients who had a visit with primary care during the evaluation period, n^a^**			
	Total number of alerts	106	59
	Unique visits or encounters	104	59
	Unique patients with alert	61	26
	Unique clinicians alerted	87	31
**Adoption (did not outright dismiss clinical decision support alert), n (%)**			
	Alerts adopted	66 (62.3)	17 (28)
	Unique patients	44 (72)	13 (1)
	Unique clinicians exposed to the alert	60 (69)	13 (41)
	Clinicians who adopted with the first alert	55 (63)	11 (35)
**Effectiveness, n (%)**			
	Alerts where BB^b^ was prescribed	15 (14.2)	0 (0)
	Unique patients where BB was prescribed	15 (25)	0 (0)
	Unique patients prescribed with first alert	13 (87)	0 (0)
	Unique patients prescribed BB by assigned primary care provider	7 (47)	0 (0)
	Unique clinicians who ever prescribed BB	14 (16)	0 (0)
	Clinicians who were attending physicians	9 (60)	0 (0)
	Clinicians who were advanced practice clinicians	3 (21)	0 (0)
	Clinicians who were a medical resident or fellow	2 (14)	0 (0)

^a^Four patients were exposed to both alerts, and 1 clinician was exposed to both alerts. One clinician prescribed a BB to 2 different patients.

^b^BB: beta-blocker.

### Clinician Interviews: Usability, Satisfaction, and Design Features Influencing Adoption

The saturation of ideas was achieved after 21 interviews that included 15 clinicians exposed to the enhanced alert and 6 exposed to the commercial alert. One clinician was exposed to both alerts and did not recall either of the exposures. A total of 40% (6/15) of clinicians exposed to the enhanced alert and none exposed to the commercial alert stated that they recalled it, either before or after being prompted with a visual reminder. In total, 24% (5/21) of clinicians preferred the commercial alert, 2 because of brevity, 2 because of the dismiss option, and 1 because of the many options available within the order set. Most clinicians (19/21; 90%) stated that they felt an alert for BBs and HFrEF should be continued. Mean SUS scores were 65.7 (SD 14.2) and 53.4 (SD 14) for the enhanced and commercial alerts, respectively. The enhanced alert had higher median Likert scale scores for the survey questions related to workflow integration (3 vs 2.5) and perceived impact on patient care (4 vs 3.5). Multimedia Appendix 4 summarizes the SUS scores and survey questions with indices commonly used to interpret SUS scores.

During the open-ended discussions, the participants identified salient design features that influenced their alert preference. In general, clinicians preferred the enhanced alert because it was easier to digest the information presented and quickly determine its purpose. Clinicians liked the use of emphasis with different font sizes, bolding, and colors to draw their attention to key aspects of the enhanced alert. Furthermore, clinicians were unfamiliar with the *express lane* terminology of the commercial alert and stated that uncertainty about the consequences of selecting this option would deter adoption. Most clinicians felt that the commercial alert needed more information, so they could evaluate the appropriateness of the recommendation for a given patient. Although it was denser, most clinicians felt that the clinical information (eg, vital signs) in the enhanced alert was necessary and preferred. With one exception, the ability to *pend* a medication order within the enhanced alert was preferred over the order set in the commercial alert. The medication order option was preferred because it required fewer *clicks*, was specific to the recommendation, and provided information regarding which medications and doses were appropriate. Table 4 summarizes the representative quotes from clinicians that distinguish between the alerts.

**Table 4:** Representative clinician quotes distinguishing between the enhanced and commercial alerts.

Description of design features referred to	Quotes referring to the enhanced alert	Quotes referring to the commercial alert
Catching attention and use of emphasis	“The color...different colors, catch our attention.” “The little heart icon gets your attention.”	—^a^
Inclusion of a dismiss option	—	“It encourages dismissal. It seems like the acknowledge reason is also a form of dismissal.”
Clarity and uncertainty	“It's much clearer in terms of what you're asking me is to order a bleeping [sic] beta-blocker, right? And you make it easy because you're clicking the most common starting doses.”	“Clicking on something where it goes to a black hole, or I don’t know where it's going, especially if there is no training. I'm less likely to click on an unknown. Like this could end up 20 different ways that ends up with 10 different screens.” “I don't like this one as much, and I think it's because when I'm reading it, immediately, I have questions popping up, and while I think, I'm kind of in a hurry. And I don't know if I want to be clicking all these things to see what this is about. So express lane that makes me think of going to a gas station for an oil change.”
Brevity and completeness of supporting information	“It gives me the pieces of information that I would want to know to make a clinical decision and then it allows me to actually make that decision. You know, to pend up an order quickly.”	“I think this is more concise so I'm more prone to read it because this one [enhanced] vomited on me.” “This is nice and simple, but perhaps it's a little too simple.”
Make it easy to do the right thing; ease of use	“Yeah, I love that you picked the 3 medicines that I should be thinking about and kind of a typical starting dose, that's great.” “Easier to use. I don't have to leave the screen.”	“A little overwhelming for like labs now, labs in 3 months, labs in 6 months, echo now, 3, 6 months. And then medications, like every medication known to mankind.”

^a^No relevant quote available

## Discussion

### Principal Findings

This study suggests that an enhanced CDS alert informed by CDS design best practices and an IS framework results in improved CDS adoption and effectiveness compared with a generic commercial alert. This conclusion is further supported by other findings related to the enhanced alert, including greater patient reach, higher usability scores, clinician-stated preference during the interviews, and the perceived impact on patient care and workflow integration. The commercial alert did not change prescribing, whereas the enhanced alert was associated with a 24% increase in BB prescriptions. Although 24% (5/21) of the interviewed clinicians preferred the commercial alert, their preference was driven by design features that were not prioritized by the majority of interview participants. Taken together, the results of this study suggest that applying the PRISM/CDS best practices approach [10] may improve the quality of care and, potentially, patient outcomes.

We achieved higher rates of adoption and effectiveness with our enhanced CDS tool than have previously been reported with other CDS tools designed to improve the prescription of similar medications for HFrEF. An RCT comparing a CDS tool with no CDS tool found no difference in effectiveness in changing HFrEF prescribing (23% vs 22%) [31], whereas another study found that a CDS tool improved prescribing by only 3.6% compared with 0.9% without a CDS (*P*=.01) [32]. These studies evaluating the effectiveness of changing HFrEF prescribing demonstrated minimal or no difference, whereas we found a 24% improvement in prescribing compared with an active control.

We identified aspects of the enhanced alert that need improvement, which developers should consider. For example, standardized drug vocabularies such as RxNorm should be used when possible and may have prevented the enhanced CDS from firing for patients with a *beta-adrenergic blocking agts* allergy. To mitigate the future risk of unintended prescriptions with the enhanced alert, we can reconsider the fundamental hard stop design. However, setting the default action of the enhanced alert to the desired change (ordering a BB) is aligned with CDS design best practices [3] and was done intentionally. Changing this design would likely minimize future instances of erroneous prescribing but might also deter effectiveness.

The reach (number of alerts, number of patients, and clinicians alerted) of the commercial alert was lower than that of the enhanced alert, which is likely because the build specifications were more constricting. Notably, the commercial alert had the following properties: (1) it required patients to have both a diagnosis of heart failure and a reduced EF, (2) it did not alert for some patients prescribed a nonevidence-based BB (ie, metoprolol tartrate), and (3) it excluded patients with an allergy to a beta-agonist, including albuterol inhalers. These 3 build specifications do not align with evidence-based clinical recommendations and limit the ability of the alert to reach the intended patients. In our instance, inaccuracies in vendor-supplied knowledge content led to poor sensitivity and false negatives. However, such inaccuracies in knowledge management could also lead to poor specificity and worsen alert fatigue, as hypothesized by others [1,5,6].

The adoption of the commercial alert was also lower. On the basis of the interview findings, the adoption of the commercial alert could be improved by applying generalizable CDS design best practices that do not require input from the local setting. For example, any clinician considering BB initiation for HFrEF needs to know the patient’s BP and heart rate. We estimate that 80% of the design decisions do not require input from the local context. Multimedia Appendix 5 describes examples of design features that do and do not require user-centered input from the local context.

Not all commercial CDS tools have the same limitations. The results of this study may have been different if the active control was a different commercial CDS tool. However, when relying on commercial CDS tools, this research highlights the need for institutions to carefully review the knowledge content and design features to ensure that they are accurate and appropriate for the local context. At a minimum, when resources are limited, institutions should review commercial CDS tools to evaluate unanticipated harm. There are advantages to relying on commercially available CDS, notably the need for fewer resources, but this may come at the cost of reduced implementation success. Similarly, there are advantages to customizing CDS for the institutional context, notably greater local ownership and implementation success, but this may come at the cost of greater resource burden. Our study demonstrates the difference accounting for the local context via an IS framework can have on implementation outcomes. By using an IS framework, CDS can be pragmatically customized to institution-specific contexts in a manner that is reproducible by other institutions and thereby generalizable.

Although adaptations to the local context may be inevitable to maximize implementation outcomes, additional efforts to share successful CDS tools across institutions are needed. Greater collaboration across institutions and repositories, such as Agency for Health care Research and Quality’s CDS Authoring Tool and Connect Repository [33], can facilitate wider dissemination of well-designed CDS tools. Furthermore, given their influence over many health systems, EHR vendors could commit to increased surveillance and updates to the knowledge and design of CDS tools. Although external vendors may be unable to customize CDS tools to the local context, they should use CDS design best practices that are generalizable (Multimedia Appendix 5) and ideally consult with content experts to optimize the accuracy of knowledge content. External vendors should be transparent in the construction of their knowledge content and technologies and, where possible, apply CDS design best practices and IS frameworks such as PRISM.

### Limitations

This study has several limitations. First, our measure of adoption aimed to identify clinicians who considered the information presented and relied on clinician responses to the alert, which can be imprecise. Similarly, our measure of effectiveness sought to capture clinicians who prescribed an evidence-based BB in response to the alert. We cannot say with certainty that the alert led to a prescribing change. It is possible that the clinician intended to prescribe the BB, and their actions were independent of the alert. However, a strength of this study is that we validated instances of BB prescriptions with chart review. Reliance on clinician-stated responses to alerts would have significantly overestimated the effectiveness of the enhanced alert. Although there are inherent limitations in our measures of adoption and effectiveness, our qualitative findings substantiate the validity of the quantitative methods.

In the initial design of this study, we planned to target 784 subjects and use generalized estimating equations to account for the within-clinic correlation in the analyses. However, due to a smaller-than-anticipated sample size and zero changes in prescribing behavior associated with the commercial alert, we needed to alter our plans. Although we were able to detect statistically meaningful differences, our small sample size warrants further research in larger populations and for different patient care scenarios. Similarly, the 21 clinicians we interviewed were not representative of all clinicians, but we did take measures to maximize credibility, transferability dependability, and confirmability of the qualitative methods [26,34]. Although the investigator (KT) who led the interviews also led the design of the enhanced CDS tool, biases were minimized by using a semistructured interview guide and documenting a priori preconceived ideas and biases. We also used a multidisciplinary approach for the thematic analysis in which an independent investigator (JS) led the coding with iterative input from 3 other investigators (JS, DM, and JK).

Finally, because much of our data were collected from the EHR, limitations inherent to secondary data sources and EHR data apply. One notable limitation is the inaccuracy and incompleteness of assigning PCPs to specific clinics within the EHR. Difficulty in accurately identifying PCP—and patient—clinic assignments prevented us from controlling for all potential cross-contamination of alert exposure. As we found, some clinicians practice at and some patients are seen at more than 1 clinic. Inaccuracy in the patient-clinic assignment also precluded us from defining the ideal denominator for reach. Furthermore, data limitations prevented us from characterizing clinician- and clinic-level characteristics that may have influenced implementation success and reporting a complete CONSORT or expanded CONSORT figure [18,35].

### Conclusions

This study suggests that applying CDS design best practices with an IS framework to CDS tools leads to meaningful improvements in patient reach, clinician adoption, and effectiveness of behavior change, as compared with some commercially available CDS tools. Future research should assess the generalization of these results and consider how this IS-based approach to CDS implementation can be adapted to rapid prototyping of CDS to expedite the creation of widely adopted, effective, and sustainable CDS.

## Appendix

Multimedia Appendix 1 Differences between vendor specifications and actual specifications for commercial.

Multimedia Appendix 2 Definitions for comorbidities of interest.

Multimedia Appendix 3 Categorization of clinician responses as an instance of adoption or not.

Multimedia Appendix 4 Usability, perceived impact, and workflow integration scores.

Multimedia Appendix 5 Examples of CDS design features that do and do not necessitate input from the local context.

Multimedia Appendix 6 General CONSORT checklist (2010).

## Abbreviations

BB: beta-blocker
BP: blood pressure
CDS: clinical decision support
CONSORT: Consolidated Standards of Reporting Trials
EF: ejection fraction
EHR: electronic health record
HFrEF: heart failure with reduced ejection fraction
PCP: primary care provider
PRISM: Practical, Robust, Implementation, and Sustainability Model
RCT: randomized controlled trial
RE-AIM: Reach, Effectiveness, Adoption, Implementation, and Maintenance
SUS: System Usability Scale
