# Supplementary material for: Applying Clinical Decision Support Design Best Practices With the Practical Robust Implementation and Sustainability Model Versus Reliance on Commercially Available Clinical Decision Support Tools: Randomized Controlled Trial
Source: JMIR Med Inform. 2021 Mar 22;9(3):e24359. doi: 10.2196/24359 (PMC8077777; doi:10.2196/24359)
Supplement: Multimedia Appendix 1 [file medinform_v9i3e24359_app1.docx]

**Appendix 1. Differences between vendor specifications and actual specifications for commercial alert build**

| **Vendor specification** | **Actual specification used** | **Rationale for change** |
| --- | --- | --- |
| Most recent EF < 40% | Most recent EF < 40% | The evidence-based indication for BB therapy is when EF is less than OR equal to 40%. Further, the vendor-specified method of configuring the logic for this did not align with how our health system stored EF’s as a structured data element, thus we used the same logic criteria for the customized and commercial for efficiency. |
| Vendor order set | Vendor order set that has been iteratively modified by the health system over time prior to this go live | Any vendor specifications for a CDS tool would be built in this manner to capitalize on prior optimizations versus revert to less ideal states |
| Trigger was inpatient discharge | Trigger was opening patient chart | Modified to fit different point in patient care process. |

BB=beta-blocker; CDS=clinical decision support; EF=ejection fraction
