# Supplementary material for: Applying Clinical Decision Support Design Best Practices With the Practical Robust Implementation and Sustainability Model Versus Reliance on Commercially Available Clinical Decision Support Tools: Randomized Controlled Trial
Source: JMIR Med Inform. 2021 Mar 22;9(3):e24359. doi: 10.2196/24359 (PMC8077777; doi:10.2196/24359)
Supplement: Multimedia Appendix 2 [file medinform_v9i3e24359_app2.docx]

| **Appendix 2. Definitions for comorbidities of interest** | |
| --- | --- |
| **Comorbidity** | **Definition** |
| Asthma | > 2 ICD9/10 codes listed as billing diagnosis |
| COPD |  |
| Atrial fibrillation |  |
| Coronary artery disease | > 2 ICD9/10 codes listed as billing diagnosis separated by at least 3 months^a^ |
| Ischemic cardiomyopathy |  |
| Non-ischemic cardiomyopathy |  |
| ^a^To prevent rule out scenarios | |
