# Supplementary material for: Applying Clinical Decision Support Design Best Practices With the Practical Robust Implementation and Sustainability Model Versus Reliance on Commercially Available Clinical Decision Support Tools: Randomized Controlled Trial
Source: JMIR Med Inform. 2021 Mar 22;9(3):e24359. doi: 10.2196/24359 (PMC8077777; doi:10.2196/24359)
Supplement: Multimedia Appendix 3 [file medinform_v9i3e24359_app3.docx]

| **Appendix 3. Categorization of clinician responses as an instance of adoption or not** | | |
| --- | --- | --- |
| **Alert** | **Adoption** | **Not adoption** |
| **Enhanced** | - Did anything other than initially order and cancel   - Selected acknowledge reason initially   - Followed recommendation | - Initially ordered then canceled medication irrespective of whether additional action was taken later unless they followed the recommendation |
| **Commercial** | - Opened express lane - Selected acknowledge reason - Followed recommendation | - Did not select open express lane or acknowledge reason |

Because the enhanced alert did not have a “dismiss” button, it was not possible to categorize dismissals as non-adoption and everything else as adoption. Therefore, the definition of adoption was defined by evaluating themes of clinician responses to the alert. These definitions of adoption were intended to capture instances in which clinicians paid attention to the information presented within the alert. Our definition of adoption considers instances in which the clinician provided some response other than finding the quickest way to dismiss the alert without digesting the information provided. For example, there is no dismiss button for the enhanced alert and the quickest way to get rid of the alert is to pend an order for metoprolol succinate. Therefore, instances in which a clinician initially pended an order for metoprolol succinate and then canceled it was categorized as non-adoption for the enhanced alert. This sequence of pending and then canceling the pended order suggests the clinician did not pay attention to the information provided and looked for the easiest way to get rid of the alert, which is to hit “accept” and pend the defaulted medication order. For both alerts, clinicians were defined as an adopter if they provided a reason they did not follow the recommendation, given that suggests they took the time to consider the information on the user interface and decide what the appropriate step should be versus hitting “dismiss” (commercial) or inadvertently pending the default medication (enhanced). Providing a comment in response to the alert was also considered an instance of adoption, irrespective of the tone of the comment, because it indicates the clinician considered the information.
