# Supplementary material for: Applying Clinical Decision Support Design Best Practices With the Practical Robust Implementation and Sustainability Model Versus Reliance on Commercially Available Clinical Decision Support Tools: Randomized Controlled Trial
Source: JMIR Med Inform. 2021 Mar 22;9(3):e24359. doi: 10.2196/24359 (PMC8077777; doi:10.2196/24359)
Supplement: Multimedia Appendix 4 [file medinform_v9i3e24359_app4.docx]

**Appendix 4. Usability, perceived impact, and workflow integration scores**

| **Alert Evaluated** | **SUS score (SD)** | | | | | | **I think the alert will fit well into my workflow** | | | **I think the alert will improve clinical decision making and patient care** | | |
| --- | --- | --- | --- | --- | --- | --- | --- | --- | --- | --- | --- | --- |
|  | Mean (SD) | Grade (A to F) | Percentile | Adjective | Acceptable | Net Promoter Scale | Median^a^ | IQR | min, max | Median^a^ | IQR | min, max |
| Enhanced (n=14) | 65.7 (14.2) | C | 41-59 | Good | Marginal | Passive | 3 | 2 | 1,5 | 4 | 1 | 2,5 |
| Commercial (n=6) | 53.2 (14) | D | 15-34 | OK | Marginal | Detractor | 2.5 | 1 | 2,3 | 3.5 | 1.75 | 2,4 |

^a^5-point Likert scale with 5=Strongly agree and 1=Strongly disagree

SUS=system usability scale

Of the 21 participants, one participant exposed to the customized alert inadvertently left one of the SUS questions incomplete, thus was unable to be scored. The single participant exposed to both alerts completed the SUS survey from the perspective of being exposed to the enhanced alert, which was their first exposure.

| **Method of interpreting SUS scores** | **Description** |
| --- | --- |
| Grade | Letter grades assigned (A+, A, A-, B+, B, B-, C+, C, C-, D, F, where A=senior performance, C=average, F=failing performance) |
| Percentile range | Compared to percentile ranks of normalized SUS scores |
| Adjective | 6-point scale of adjective words (best imaginable, excellent, good, OK, poor, worst imaginable) |
| Acceptability | 3-point scale of adjective words (acceptable, marginal, not acceptable) |
| Net Promoter Scale | Describes likelihood of end-user to recommend using the tool (promoter, passive, detractor) |
