# Supplementary material for: Applying Clinical Decision Support Design Best Practices With the Practical Robust Implementation and Sustainability Model Versus Reliance on Commercially Available Clinical Decision Support Tools: Randomized Controlled Trial
Source: JMIR Med Inform. 2021 Mar 22;9(3):e24359. doi: 10.2196/24359 (PMC8077777; doi:10.2196/24359)
Supplement: Multimedia Appendix 5 [file medinform_v9i3e24359_app5.docx]

**Appendix 5. Examples of CDS design features that do and do not necessitate input from the local context**

| **Features that can be addressed without local input^a^** | **Features that require local input** |
| --- | --- |
| Translation of evidence into structured logic or algorithms | Consideration of local influences such a state/regional regulations, adapting evidence to meet the needs of local protocols, adapting recommendations for local formularies |
| Presentation of pertinent information needed to evaluate appropriateness of the CDS recommendation | Identification of appropriate discrete data elements to inform the logic or algorithm |
| Transparency and comprehensiveness of response options that make it easy to understand consequences and do not force a response that is unaligned with the end-user’s intentions | Workflow integration |
| Use of different font sizes, colors, spacing that makes it easy to understand and digest the information |  |
| Provide actionable and specific response options to act on the CDS recommendation |  |
| Use clear and transparent language that is universally understood |  |

^a^Design features that do not require local input will still benefit from local input from actual end-users. There are multiple approaches to addressing design features which can be influenced by end-user preferences.
